# Exploring the injury severity of unlicensed powered two- and three-wheeler drivers in two-vehicle crashes in China

**DOI:** 10.1038/s41598-025-88896-3

**Published:** 2025-04-06

**Authors:** Peixiang Xu, Fulu Wei, Dong Guo, Yongqing Guo, Lizu Sun, Chuan Liu, Bin Zhou

**Affiliations:** 1 School of Transportation and Vehicle Engineering, Shandong University of Technology, Zibo, 255000 China; 2 State Key Lab of Intelligent Transportation System, Beijing, 100000 China

**Keywords:** Two-vehicle crash, Powered two- and three-wheeler, Unlicensed driver, Machine learning, Engineering, Risk factors

## Abstract

Large presence of unlicensed powered two- and three-wheeler (PTW) drivers in China pose a significant threat to road safety. In this study, a customized Deep Forest Model (DF-ptw) is constructed to investigate the effect of unlicensed PTW drivers on crash severity in two-vehicle crashes, using a recent 3-year historical crash data. SHapley Additive explanation (SHAP) and Partial Dependence Plot (PDP) analysis reveal that unlicensed motorcyclists are significantly more likely to suffer serious injuries in two-vehicle crashes compared to unlicensed auto-rickshaw drivers. Additionally, factors such as drunk driving, fatigued driving, and being an unlicensed driver over the age of 53 notably elevate the risk of serious injury or death, with unlicensed motorcyclists being disproportionately affected. Moreover, self-employed unlicensed PTW drivers face a higher probability of serious injury or fatality in crashes compared to farmers, blue-collar, and white-collar workers. Unlicensed PTW drivers are also more susceptible to severe or fatal injuries on national and provincial roads, in low visibility conditions, during late-night hours, on non-separated roads, and at dusk or dawn. Based on these findings, this study proposes to reduce the frequency and severity of crashes involving unlicensed PTW drivers by focusing on more stringent eligibility checks, increasing safety awareness, and implementing advanced safety measures.

## Introduction

### Background

Powered two- and three-wheelers (PTWs), such as motorcycles, and auto-rickshaws have high accessibility, which is important for transportation, complementing, competing with, and supplementing other modes especially in developing countries^1^. However, PTWs often mix with four-wheeled vehicles in traffic and are more prone to loss of control, increasing their likelihood of being involved in serious two-vehicle crashes^2^. The percentage of fatalities for PTWs is 27% in China, which means almost 70,000 people died in PTW-involved crashes per year^3^. Additionally, there are many unlicensed PTW drivers in China. The lack of professional driver training significantly increases road safety risks.

In China, a highly permissive vehicle market enables buyers to acquire PTWs without possessing a driver’s license. The abundance of second-hand PTWs with existing license plates in the unregulated market al.lows individuals without licenses to freely drive on roads without intervention from traffic police^4^. Additionally, drivers without specialized training are often unfamiliar with traffic laws, making them more likely to be involved in two- or multiple-vehicle collisions, especially at intersections and when other drivers are not paying attention to them^5,6^. Therefore, it is necessary to explore the impact of unlicensed PTW drivers on crash severity in two-vehicle crashes.

### A brief review of past studies

This section provides a brief review of prior research on factors affecting the severity of two-vehicle crashes involving various PTW models. It then shifts focus to the impact of driving PTWs without a license on crash outcomes and discusses accident analysis methods pertinent to this study.

PTW drivers tend to suffer more severe injuries in crashes than four-wheelers. Consequently, researchers have conducted numerous studies over the years on the risks and severity of crashes affecting PTW safety. Previous studies conducted in various regions have demonstrated that several factors significantly impact the severity of PTW driver injuries, including PTW type, age, gender, roadway type, speed limits, time of day, and natural environment^7,8^. However, differences between PTW models can lead to variations in how the same influencing factor affects crash severity.

For motorcyclists, older riders (age > 65), alcohol involved, or not wearing a helmet significantly affect serious injury outcomes^9,10^. In two-vehicle crashes, the crash partner also influences the crash severity. Motorcycles are more likely to be involved in serious crashes with trucks due to the large number of blind spots in the truck’s field of vision^11^. The location (e.g., intersection) and type of crash (e.g., rear-end) also contribute to the severity of two-vehicle crashes^12,13^. In addition, several external environmental factors can significantly impact the outcome of serious injuries, such as slippery road surfaces, lack of lighting at night, and poor visibility^14^.

Auto-rickshaws involved in two-vehicle crash differ somewhat from motorcycles and mopeds. Auto-rickshaws have a lower risk of serious crashes in two-vehicle crashes compared with motorcycles. The enclosure provides safety to its occupants in case of a crash with a car or a heavier vehicle^15^. Furthermore, most auto-rickshaws do not have seat belts, and in a crash, the driver is often thrown out from the vehicle, resulting in head injuries^16^. Additionally, a study from Pakistani identified several factors that exacerbate the severity of crashes involving auto-rickshaws. These factors include driving during the daytime, weekdays, off-peak periods, and under clear weather conditions^8^.

Regardless of the type of PTW, studies have shown that unlicensed driving exacerbates the risk of crashes, particularly in developing countries. These groups are more likely to include farmers in suburban or rural areas. Factors such as economic conditions, accessibility and availability of driver licensing and training, and age limitations lead these groups to drive without a license^17^. Dangerous driving behaviors, such as driving under the influence of alcohol, driving at night, speeding, and running red lights, often accompany unlicensed driving^18^. However, most current research has focused solely on unlicensed driving as a factor affecting crash severity, lacking in-depth research on this group.

In addition, in recent years the advancement of Machine learning (ML) algorithms has led to their increased adoption in traffic safety research^19^. ML is more frequently used as a prediction tool than traditional statistical methods. However, the two approaches share commonalities. Both ML and statistical methods aim to improve forecasting accuracy by minimizing a loss function^20^. ML methods are also more computationally demanding, relying heavily on computer science, which places them at the intersection of statistics and computer science^21^. Researchers favor a type of tree model with a branching structure based on feature space partitioning. This model is preferred because it does not require specific measurements, comprises multiple rules, can handle both numerical and categorical data, and offers high interpretability. Although statistical modeling and machine learning methods follow different methodological streams for prediction, the identified risk factors are largely consistent^22^.

### Study objective

The reviewed studies indicate that unlicensed PTW driving can result in severe crash outcomes, particularly in developing countries. However, limited attention has been given to understanding the specific impacts of unlicensed PTW drivers, especially in the context of crash severity. Key aspects such as driver status, employment status, and age distribution have been underexplored in relation to these drivers. This study, therefore, aims to fill this gap by deeply investigating the impact of unlicensed PTW drivers on two-vehicle crashes in China.

A key novel aspect of this research lies in the use of a customized Deep Forest (DF-ptw) model, which uniquely integrates multiple tree-based algorithms to capture complex patterns in accident data. This model enables a more nuanced understanding of how different factors and their interactions contribute to the severity of crashes involving unlicensed PTW drivers. By customizing the model specifically for PTW-related crashes, we provide new insights into the underlying causes of these crashes, contributing to the existing body of knowledge.

To analyze the effects of individual factors and their interactions on crash severity, the study employed SHAP (Shapley Additive Explanations) and PDP (Partial Dependence Plots) interpretation tools. SHAP quantifies the impact of individual variables on the model’s predictions, providing an understanding of how each feature influences the likelihood of severe injury outcomes. PDP allows us to visualize the marginal effect of one or more features on the predicted outcome, helping to identify key variables that may break the accident chain and reduce crash severity.

## Data processing and description

**Fig. 1:**
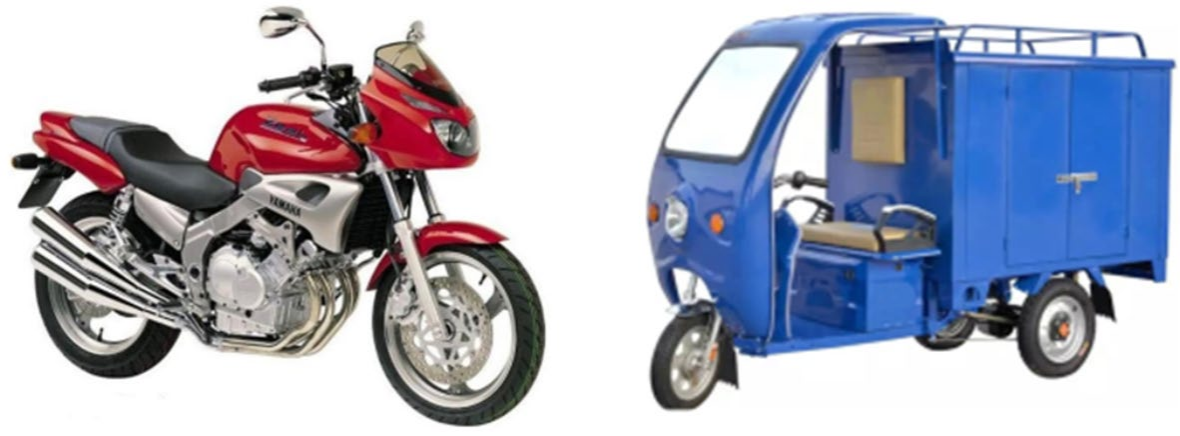
Common types of unlicensed PTWs in China: motorcycle (left), and auto-rickshaw (right).

The data for this study are obtained from unlicensed PTW drivers involved in two-vehicle crashes in Shandong Province, China, from 2020 to 2022, a total of 5777 unlicensed PTW drivers involved in two-vehicle crashes are obtained after data processing. The data are provided by the Center for Accident Research in Zibo (CARZ). The classification of PTWs in this study is based on the public safety industry standards promulgated by the People’s Republic of China in 2019: Road traffic management – Types of motor vehicles. According to these standards, PTWs are categorized into three subcategories, examples as shown in the Fig. 1.

1. Motorcycle: motorcycles with a maximum design speed greater than 50 km/h
2. Auto-rickshaw: motorcycles equipped with two rear wheels symmetrically distributed with the front wheel

In two-vehicle crashes, the crash partner involving minibuses, light trucks, medium trucks, large buses, and heavy trucks. These vehicles are common in the transportation system. The conservation of momentum in a crash places smaller vehicles at a disadvantage when the crash partner is heavier. The difference in injury severity (DIS) between an unlicensed PTW driver and a crash partner driver (CPD) in a two-vehicle crash can be defined as,

1 $$DIS={S_{NPTWD}} - {S_{CPD}}$$

Where $\:{S}_{NPTWD}$ and$\:\:{S}_{CPD}$ represent the injury severity of unlicensed PTW driver and crash partner driver, respectively, with values ranging from 0 to 3 integers, indicating no injury, slight injury, serious injuries, and fatality, respectively. And, where $\:DIS$ indicates the degree of difference in injuries between the two vehicles involved in the same crash. A positive value indicates that the unlicensed PTW driver is more severely injured than the crash partner driver, while a negative value suggests the opposite. A value of zero signifies that there is no significant difference in the degree of injury between the drivers of the two vehicles. The statistics on $\:DIS$ are displayed in Table 1 basing on original data.

**Table 1:** Difference in injury severity statistics between PTW drivers and other drivers.

Crash partner	Count	DIS						
		−3	−2	−1	0	1	2	3
Minibus	3465	0.1%	0.1%	0.9%	8.1%	71.5%	5.7%	13.7%
Light truck	455	-	0.2%	0.4%	8.1%	64.6%	5.5%	21.1%
Medium truck	194	-	-	2.1%	6.7%	53.6%	9.8%	27.8%
Large bus	118	-	-	0.9%	6.8%	56.8%	11.0%	24.6%
Heavy truck	1545	-	-	0.8%	4.0%	51.7%	6.9%	36.5%

Table 1 Illustrates that the majority of unlicensed PTW drivers experienced injury severity one level higher than the drivers of crash partner. When the crash partner is a Minibus, 71.5% of cases result in $\:DIS$=1; 64.6% for light truck; 53.6% for medium truck; 56.8% for large bus; 51.7% for heavy truck. PTWs face significantly higher vulnerability within the transportation system^23^. Driver injuries can reflect the immediate consequences of crashes, providing a tangible measure of severity^24^. Therefore, the injury severity of unlicensed PTW drivers is used to measure the severity of two-vehicle crashes. The original dataset categorized injuries into four severities: no injury (5.6%, 344 pieces of data), slight injury (66.6%, 3848 pieces of data), severe injury (5.8%, 335 pieces of data), and fatal injury (21.6%, 1250 pieces of data). A fatal injury is defined as a driver passing away within seven days. We group no and slight injuries together, labeled as NS injuries, to mitigate the influence of under-reporting on model performance. And, the severe injury and fatal injury are combined and recorded as serious or fatal Injury (SFI). The injury severity of unlicensed PTW drivers is defined as the response variable, including NS and SFI. Moreover, potential contributing factors are modeled as explanatory variables in table 2.

**Table 2:** Details of variables

Variables (encoding)	Injury severity count of the unlicensed PTW driver		
	NS	SFI	Total
	4192 (72.6%)	1585 (27.4%)	5777
**1. PTW-related characteristics**			
1.1 PTW type (Categorical)			
Motorcycle = 0	3649 (73.0%)	1350 (27.0%)	4999
Auto-rickshaw = 1	543 (69.8%)	235 (30.2%)	778
1.2 Unlicensed PTW driver gender (Categorical)			
Male = 0	3832 (72.5%)	1450 (27.5%)	5282
Female = 1	360 (72.7%)	135 (27.3%)	495
1.3 Unlicensed PTW driver age (Continuous, from 15 to 83 years)			
18–35	997 (78.7%)	270 (21.3%)	1267
36–53	1844 (73.9%)	650 (26.1%)	2494
54–70	1182 (67.9%)	559 (32.1%)	1741
>70	169 (61.5%)	106 (38.5%)	275
1.4 Unlicensed PTW driver employment status (Categorical)			
Farmer = 0	2276 (72.2%)	875 (27.8%)	3151
Blue-collar = 1	226 (66.3%)	115 (33.7%)	341
White-collar = 2	234 (77.2%)	69 (22.8%)	303
Self-employed = 3	1456 (73.5%)	526 (26.5%)	1982
1.5 Driving behavior of unlicensed PTW driver (Categorical)			
No abnormal behavior = 0	2166 (83.9%)	415 (16.1%)	2581
Drunk driving = 1	609 (36.8%)	1044 (63.2%)	1653
Fatigue driving = 2	168 (67.2%)	82 (32.8%)	250
Furious driving = 3	701 (98.3%)	12 (1.7%)	713
Distracted driving = 4	548 (94.5%)	32 (5.5%)	580
**2. Crash partner (CP) driver (CPD)-related characteristics**			
2.1 CP type (Categorical)			
Minibus = 0	2791 (80.5%)	674 (19.5%)	3465
Light truck = 1	333 (73.2%)	122 (26.8%)	455
Medium truck = 2	119 (61.3%)	75 (38.7%)	194
Large bus = 3	75 (63.6%)	43 (36.4%)	118
Heavy truck = 4	874 (56.6%)	671 (43.4%)	1545
2.2 CPD gender (Categorical)			
Male = 0	3746 (71.3%)	1509 (28.7)	5255
Female = 1	446 (85.4%)	76 (14.6%)	522
2.3 CPD age (Continuous, from 18 to 73 years)			
18–35	1686 (74.2%)	586 (25.8%)	2272
36–53	2227 (71.2%)	902 (28.8%)	3129
>54	279 (74.2%)	97 (25.8%)	376
**3. Roadway and crash characteristics**			
3.1 Road functional class (Categorical)			
National and provincial road = 0	1305 (66.1%)	668 (33.9%)	1973
Urban road = 1	2037 (79.4%)	529 (20.6%)	2566
Rural road = 2	850 (68.7%)	388 (31.3%)	1238
3.2 Road condition classification (Categorical)			
Motorway = 0	3118 (73.5%)	1124 (26.5%)	4242
Non-motorway = 1	521 (71.9%)	204 (28.1%)	725
Mixed traffic lane = 2	553 (68.3%)	257 (31.7%)	810
3.3 Roadway alignment (Categorical)			
Planar linear = 0	3487 (73.1%)	1281 (26.9%)	4768
Bend road or rampway = 1	705 (69.9%)	304 (30.1%)	1009
3.4 Crash position (Categorical)			
Road section = 0	2632 (72.1%)	1019 (27.9%)	3651
Intersection with signal control = 1	828 (73.5%)	299 (26.5%)	1127
Intersection without signal control = 2	732 (73.3%)	267 (26.7%)	999
3.5 Physical separation of the road (Categorical)			
Non-separation = 0	2984 (71.7%)	1177 (28.3%)	4161
Only central separation (type A) = 1	803 (70.1%)	342 (29.9%)	1145
Only motorway and non-motorway separation (type B) = 2	167 (84.8%)	30 (15.2%)	197
Type A and B = 3	238 (86.9%)	36 (13.1%)	274
3.6 Road surface condition (Categorical)			
Dry = 0	3942 (72.9%)	1464 (27.1%)	5406
Surface gathered water = 1	250 (67.4%)	121 (32.6%)	371
3.7 Crash type (Categorical)			
Head-on crash = 0	652 (75.0%)	217 (25.0%)	869
Rear-end crash = 1	539 (62.7%)	320 (37.3%)	859
Side impact = 2	2855 (74.5%)	978 (25.5%)	3833
Fender-bender = 3	146 (67.6%)	70 (32.4%)	216
**4. Environment characteristics**			
4.1 Month (Categorical, from January = 1 to December = 12)			
Spring	1121 (72.3%)	430 (27.7%)	1551
Summer	860 (73.4%)	312 (26.6%)	1172
Autumn	1105 (71.1%)	449 (28.9%)	1554
Winter	1106 (73.7%)	394 (26.3%)	1500
4.2 Week (Categorical, from Monday = 1 to Sunday = 7)			
Weekday	3041 (73.2%)	1114 (26.8%)	4155
Weekend	1151 (71.0%)	471 (29.0%)	1622
4.3 Crash time (Categorical, from (0:00–1:00) = 0 to (23:00–24:00) = 23)			
Day (9:00–16:59)	1793 (74.4%)	617 (25.6%)	2410
Peak (7:00–8:59); (17:00–19:59)	1259 (74.7%)	427 (25.3%)	1686
Night (20:00–6:59)	1140 (67.8%)	541 (32.2%)	1681
4.4 Weather (Categorical)			
Clear = 0	3693 (73.1%)	1362 (26.9%)	5055
Fog = 1	297 (70.0%)	127 (30.0%)	424
Rain and snow = 2	202 (67.8%)	96 (32.2%)	298
4.5 Visibility (Categorical)			
Over 200 m = 0	2435 (75.2%)	802 (24.8%)	3237
100–200 m = 1	620 (75.9%)	197 (24.1%)	817
50–100 m = 2	609 (66.0%)	314 (34.0%)	923
Below 50 m = 3	528 (66.0%)	272 (34.0%)	800
4.6 Lighting condition (Categorical)			
Daylight = 0	2717 (74.2%)	944 (25.8%)	3661
Dusk and dawn = 1	200 (66.2%)	102 (33.8%)	302
Dark with street lamp = 2	660 (76.1%)	207 (23.9%)	867
Dark without street lamp = 3	615 (64.9%)	332 (35.1%)	947

## Methods

**Fig. 2:**
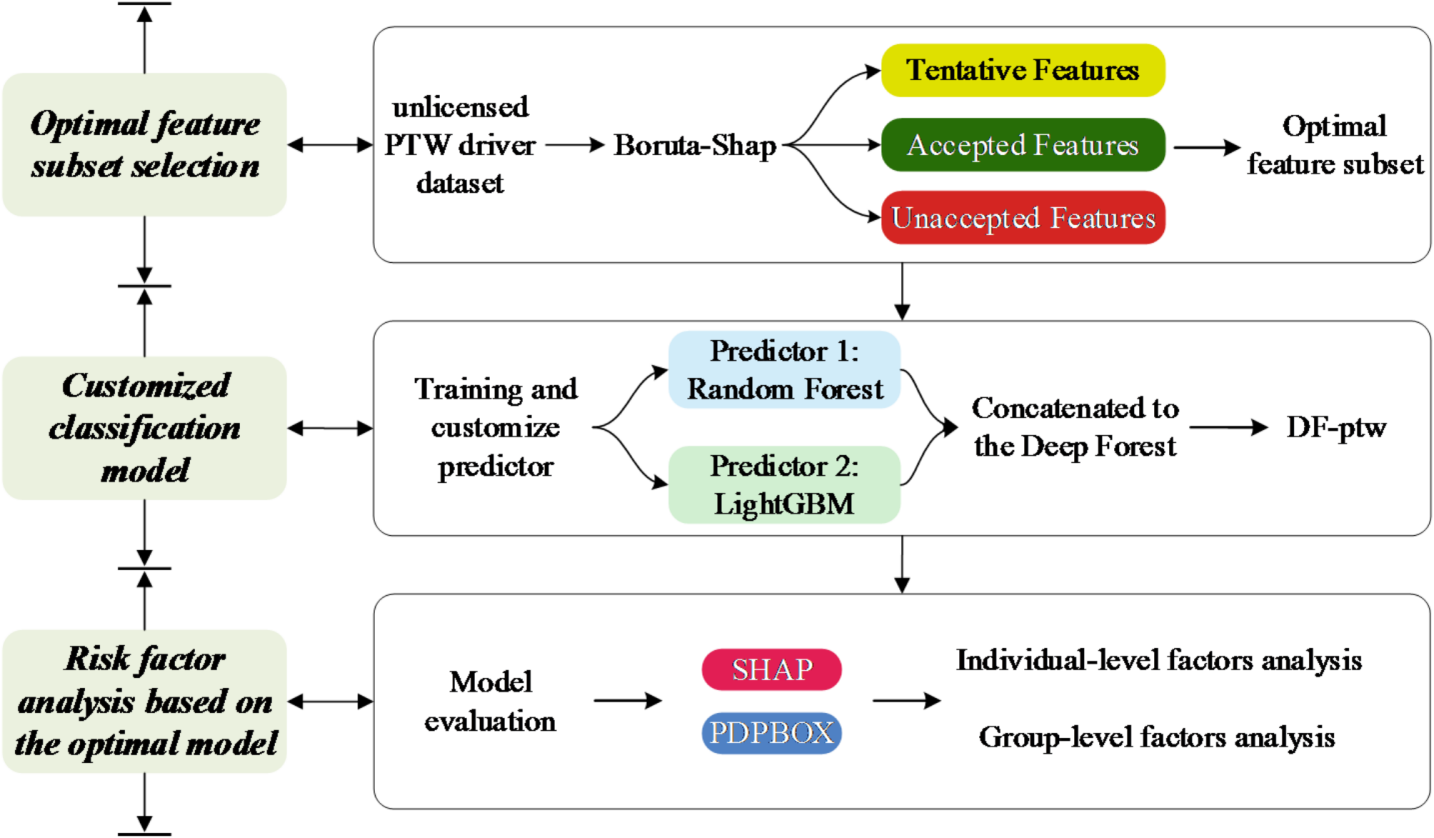
A flow chart of this study.

This study accomplishes the prediction of response variables by constructing Machine Learning classification model. The response variable, injury severity, is characterized as a categorical variable, making it suitable for prediction through classification models. The classification model outputs discrete categories that are easily understood and interpreted^25^. Additionally, the explanatory variables encompass both discrete factors and continuous factors. Classification models adeptly handle these mixed features, effectively mapping them into discrete severity categories^26^. The technical route of this study is shown in Fig. 2.

### Optimal feature subset selection

Due to the large number of candidate features, it is necessary to perform feature selection on the explanatory variables to remove redundant features^27^. Feature selection helps in identifying the most significant factors affecting injury severity, aiding in the development of targeted interventions and policies to improve road safety.

This study utilizes the Boruta-Shap algorithm classifying features as accepted, unaccepted, or tentative. It is an extension of the Boruta feature selection method that incorporates SHAP values for feature importance^28^. It sets the threshold by using the SHAP value of the shaded feature. The shadow feature $\:{X}_{i}^{shadow}$ is created by duplicating the unlicensed PTW driver dataset $\:X$ and shuffling the values within each feature $\:{X}_{i}$. Combine the original features $\:X$ with shadow feature $\:{X}^{shadow}$ to form the extended dataset$\:\:{X}^{extended}$. Calculate the SHAP value of each feature based on dataset $\:{X}^{extended}$. SHAP values show the importance of each feature:

2 $${\emptyset _i}=\sum\limits_{{S \subseteq N\left\{ i \right\}}} {\frac{{\left| S \right|!(n - \left| S \right| - 1)!}}{{n!}}} \left[ {v(S \cup \left\{ i \right\}) - v\left( S \right)} \right]$$

where $\:{\varnothing\:}_{i}$ denotes the contribution of the $\:i\text{-}th$ feature, $\:N$ is the set of all features, $\:S$ is the subset of $\:N$ with feature $\:i$, and $\:v\left(S\right)$ is the prediction result of $\:S$. Compare the SHAP value distribution of feature with the maximum SHAP value distribution of the shadow features using the t-statistic:

3 $$t=\frac{{{\mu _j} - {\mu _{\hbox{max} \_shadow}}}}{{\sqrt {\frac{{\sigma _{j}^{2}}}{n}+\frac{{\sigma _{{\hbox{max} \_shadow}}^{2}}}{n}} }}$$

Where $\:{\mu\:}_{j}$ and $\:{\sigma\:}_{j}$ is the mean and standard deviation of $\:{\varnothing\:}_{i}$ respectively for each feature $\:j$, $\:{\mu\:}_{\text{m}\text{a}\text{x}\_shadow}$ and $\:{\sigma\:}_{\text{m}\text{a}\text{x}\_shadow}$ is the maximum mean and standard deviation SHAP value among shadow features. Based on the p-value from the t-test: if $\:p<0.05$, the feature is considered accepted; if $\:p>0.05$, the feature is either unaccepted, or tentative. In this study, accepted features and tentative features are used as the optimal feature subset.

### Customized deep forest model

**Fig. 3:**
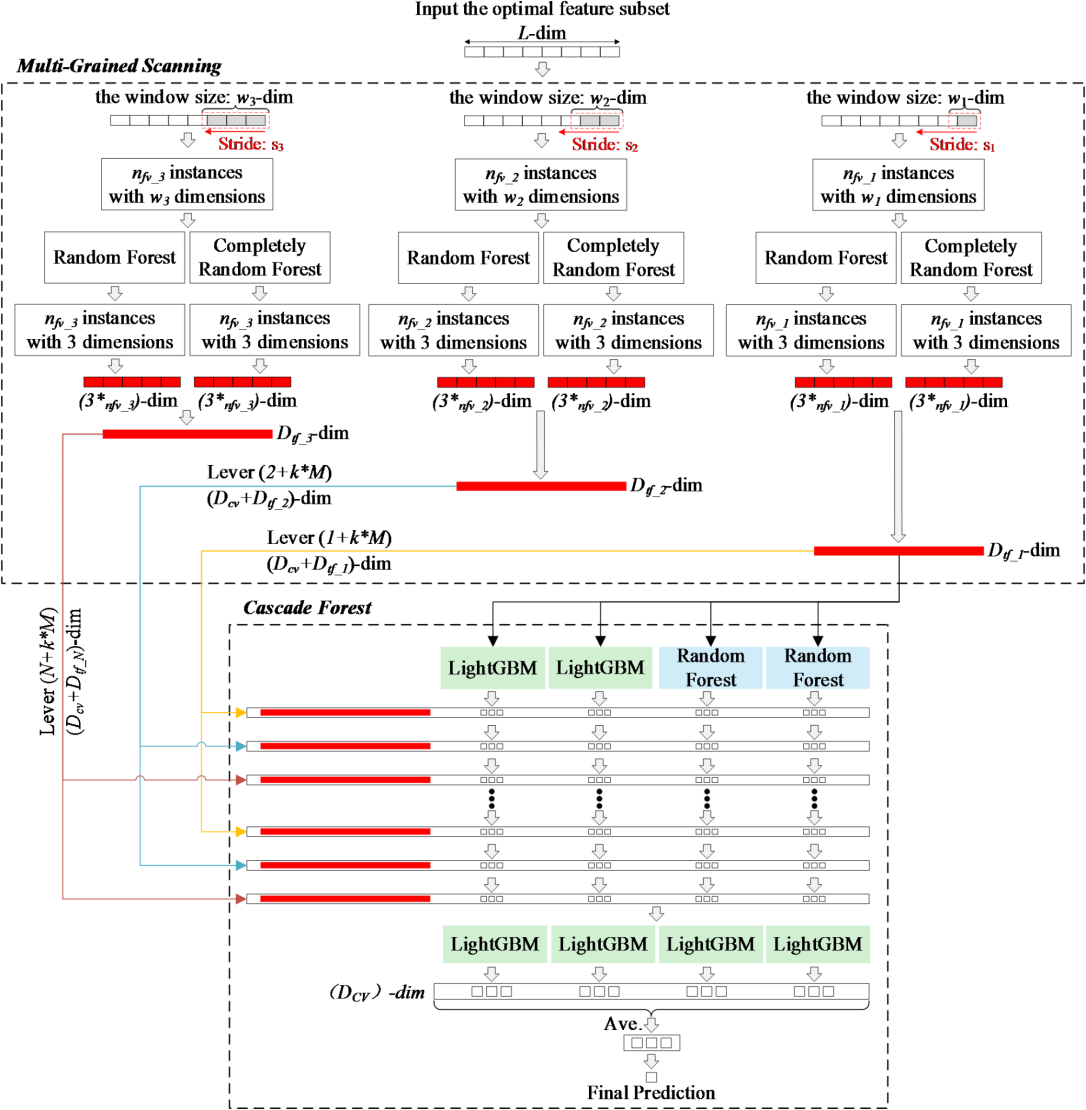
Customized Deep Forest structure.

The Deep Forest involve the Multi-grained Scanning and the Cascade Forest^29^. In this study, Random Forest and Light Gradient Boosting Machine (LightGBM), are trained using optimal feature subsets as customized predictors. The Deep Forest structure customized in this study is shown in Fig. 3.

#### Multi-grained scanning

Multi-Grained Scanning is designed to enhance feature representation by capturing patterns at different granularities^29^. It helps in extracting more informative features, especially from the high-dimensional crash data used in this study. The Multi-Grained Scanning is shown in Fig. 3. The optimal feature subset with L dimensional features and C dimensional response variable (NS and SFI) is divided into 3 sub-instances using sliding windows of different dimensions. Each sliding window uses stride $\:{s}_{i}$ extracting a local region $\:{w}_{i}\text{-}dim$ of the input data. Then the number of features vector $\:{n}_{fv\_i}$ of per sub-instance can be defined as,

4 $${n_{fv\_i}}=\frac{{L - {w_i}}}{{{s_i}}}+1$$

Next, all the $\:{n}_{fv\_i}$ extracted from the same $\:{w}_{i}\text{-}dim$window will be used to train a Completely-Random Forest and a Random Forest. The use of the Completely-Random Forest and the Random Forest increases the diversity of the base model to reduce the risk of overfitting and improve the effectiveness of the Multi-grained Scanning^30^. Then the class vectors are generated and concatenated as transformed features. The dimension of transformed feature vector $\:{D}_{tf\_i}$ corresponding to the original $\:L\text{-}dim$ can be defined as,

5 $${D_{tf\_i}}=2 * C * {n_{fv\_i}}$$

Finally, the $\:i\text{-}th$ transformed feature is used to train the $\:j\text{-}th$ level of the Cascade Forest, where $\:j$ and $\:i$ satisfy the following relationship:

6 $$j=i+k * M$$

#### Cascade forest

The Cascade Forest structure is the core architecture of Deep Forest that enables deep learning through a hierarchical, layer-by-layer processing of features^31^. It allows the model to progressively refine and improve its feature representation and predictions. The cascade layer of a traditional Cascade Forest consists of four base predictors: two Completely Random Forest and two Random Forest. The final prediction is the average of each base predictor’s results^32^. In this study, the trained Random Forest and LightGBM are used as predictors to construct the cascade layer of the Cascade Forest. Introducing the two types of predictors enhances the model’s flexibility, robustness, and performance^33^. And the LightGBM is then stacked as a meta-learner in the$\:N\text{+}1\text{-}th$ layer to build the Cascade Forest. The Cascade Forest structure shown in Fig. 3.

The transformed $\:{D}_{tf\_1}\text{-}dim$ features generated by the Multi-grained Scanning are used as the input dataset for the level 0 of the Cascade Forest. Each predictor estimates the class distribution by calculating the percentage of different classes of training $\:{D}_{tf\_1}\text{-}dim$ features examples and then averaging these percentages across all trees in the predictor. The average estimate of class distribution on the $\:{D}_{tf\_1}\text{-}dim$features dataset for the Random Forest predictor can be defined as^34^,

7 $$H({D_{tf\_1}})=\frac{1}{K}\sum\limits_{{k=1}}^{K} {{\xi _k}{h_k}({D_{tf\_1}})}$$

where, $\:k$ represents the number of decision trees integrated into the Random Forest $\:k\text{=}\text{1,2},\:\dots\:,K$. The $\:{h}_{k}\left({D}_{tf\_1}\right)$ represents the output of the $\:k\text{-}th$ decision tree for the $\:{D}_{tf\_1}\text{-}dim$ features dataset, $\:{\xi\:}_{k}$ is the weight of the $\:k\text{-}th$ decision tree. And, the average estimate of class distribution on the $\:{D}_{tf\_1}\text{-}dim$ features dataset for the LightGBM predictor can be defined as,

8 $$Obj=\left[ {\sum\limits_{{i=1}}^{N} {L\left( {{y_i},{{\hat {y}}^{(k - 1)}}+p} \right)} } \right]+\frac{1}{2}\lambda {P^2}+\gamma T$$

where, $\:\text{L}$ is the loss function $\:N$, is the number of samples $\:{D}_{tf\_1}\text{-}dim$ s features, $\:{y}_{i}$ represents the true value of the $\:i\text{-}th$ label$\:{\widehat{y}}^{\left(t\text{-}1\right)}$, is the predicted value from the previous decision tree. The $\:P$ is the predicted value of the $\:t\text{-}th$ decision tree, $\:T$ is the total number of nodes in the $\:t\text{-}th$ tree. And, both $\:\lambda\:$ and $\:\gamma\:$ are hyperparameters that are used to control the stride versus preventing overfitting, respectively. The estimated class distribution forms a $\:{D}_{cv\_1}$ dimensional class vector corresponding to the $\:{D}_{tf\_1}$ dimensional transformed feature vector.

Next, in the Cascade Forest structure, except for the level 0, each level is the combination of the original feature vector with the augmented $\:{D}_{tf\_1}\text{-}dim$ features vector generated by the previous layer. the $\:{D}_{cv\_1}$ dimensional class vector produced by the level 0 is concatenated with the $\:{D}_{tf\_1}\text{-}dim$ features vector to be input to the level 1 of the Cascade Forests. Similarly, the $\:{D}_{cv}$ dimensional class vector produced by the $\:\left(j\text{-}1\right)\text{-}th$ level will be concatenated with the $\:{D}_{tf\_i}\text{-}dim$ features vector to be input to the $\:j\text{-}th$level of the Cascade Forests until the convergence of validation performance^35^.

9 $${D_{{\text{cv}}}}={N_{rf}} * C$$

Finally, as the layers continue to be stacked, the valid information in the features is continuously enhanced. When the final layer is reached, the $\:{D}_{cv}$ dimensional class vector will no longer be combined with the $\:{D}_{tf\_i}\text{-}dim$ features vector. And, the meta learner- LightGBM models are used to construct each level. At each node splitting, the feature with the best Gini value in the $\:\sqrt{({D}_{cv}+{D}_{tf\_i})}$ randomly selected features are chosen for splitting. The final class vector $\:{D}_{last}$ contains the probability of classifying the current sample, where the class with the highest probability $\:{MAX(D}_{last})$ is the Cascade Fores’s estimate.

### Performance metrics

To assess the predictive performance of the DF-ptw, this study uses four evaluation metrics: $\:Accuracy$, $\:{Precision}_{macro}$, $\:{Recall}_{macro}$, and $\:{F}_{1}\text{-}{score}_{marco}$. The response variables in this study are 2-categorical: NSI and SFI. And, for an overall analysis of a classifier, we use macro average of $\:Precision$, $\:Recall$, and $\:{F}_{1}\text{-}score$.

The $\:Accuracy$ measures the proportion of correctly classified cases from the total number of objects in the dataset and can be calculated from Eq. 10. The $\:{Precision}_{macro}$ calculates the precision for each class separately and then takes the average of those values and can be calculated from Eq. 11. The $\:{Recall}_{macro}$ is the fraction of instances in a class that the model correctly classified out of all instances in that class and can be calculated from Eq. 12. And, the is $\:{F}_{1}\text{-}{score}_{marco}$ Harmonic mean of $\:{Precision}_{macro}$ and $\:{Recall}_{macro}$ (refer to Eq. 13).

10 $$A{\text{ccuracy}}=\frac{{{\text{Correct predictions}}}}{{{\text{All predictions}}}}$$

11 $$\Pr ecisio{n_{macro}}=\frac{{\frac{{T{P_{NS}}}}{{T{P_{NS}}+F{P_{NS}}}}+\frac{{T{P_{SFI}}}}{{T{P_{SFI}}+F{P_{SFI}}}}}}{2}$$

12 $$\operatorname{Re} cal{l_{macro}}=\frac{{\frac{{T{P_{NS}}}}{{T{P_{NS}}+F{N_{NS}}}}+\frac{{T{P_{SFI}}}}{{T{P_{SFI}}+F{N_{SFI}}}}}}{2}$$

13 $${F_1} - scor{e_{macro}}=2 * (\frac{{\Pr ecisio{n_{macro}} * \operatorname{Re} cal{l_{macor}}}}{{\Pr ecisio{n^{ - 1}}_{{macro}}+\operatorname{Re} cal{l^{ - 1}}_{{macro}}}})$$

### Interpretability of model results

In this study, SHAP and PDP are used to explain the effect of factors on the predicted results of the model. The goal of SHAP is to explain the prediction of an instance $\:{x}_{i}$ by computing the contribution of each feature to the prediction. The core idea of using the SHAP summary plot in this study is to utilize Shapley values, combining feature importance with feature effects, to show the relationship between the value of a feature and its impact on the prediction. The Shapley value of a feature value is its contribution to the payout, weighted and summed over all possible feature value combinations:

14 $${\emptyset _j}(val)=\sum\limits_{{S \subseteq \left\{ {1,\cdots,p} \right\}}} {\frac{{\mid S \mid !(p - \mid S \mid - 1)!}}{{p!}}(va{l_x}(S \cup \left\{ j \right\}) - va{l_x}(S))}$$

where $\:S$ is a subset of the features used in the model, $\:x$ is the vector of feature values of the instance to be explained, $\:p$ the number of features, and $\:{val}_{x}\left(S\right)$ is the prediction for feature values in set $\:S$.

The partial dependence plot (PDP) shows the marginal effect one or two features have on the predicted outcome^36^. The partial dependence function is defined as:

15 $${\hat {f}_S}({x_S})={E_{{X_C}}}\left[ {\hat {f}({x_S},{X_C})} \right]=\int {\hat {f}({x_S},{X_C})d} {\rm P}({X_C})$$

The $\:{x}_{S}$ represent the features for which the partial dependence function should be plotted, while $\:{X}_{C}$ denote the other features used in the machine learning model $\:\widehat{f}$, which are here treated as random variables. The feature(s) in $\:S$ are those for which the effect on the prediction is of interest. The feature vectors $\:{x}_{S}$ and $\:{X}_{C}$ together constitute the total feature space $\:X$. Partial dependence functions operate by marginalizing the machine learning model output over the distribution of the features in set $\:C$, thus illustrating the relationship the relationship between the features in set $\:S$ and the predicted outcome.

## Results and discussion

### Model evaluation

**Fig. 4:**
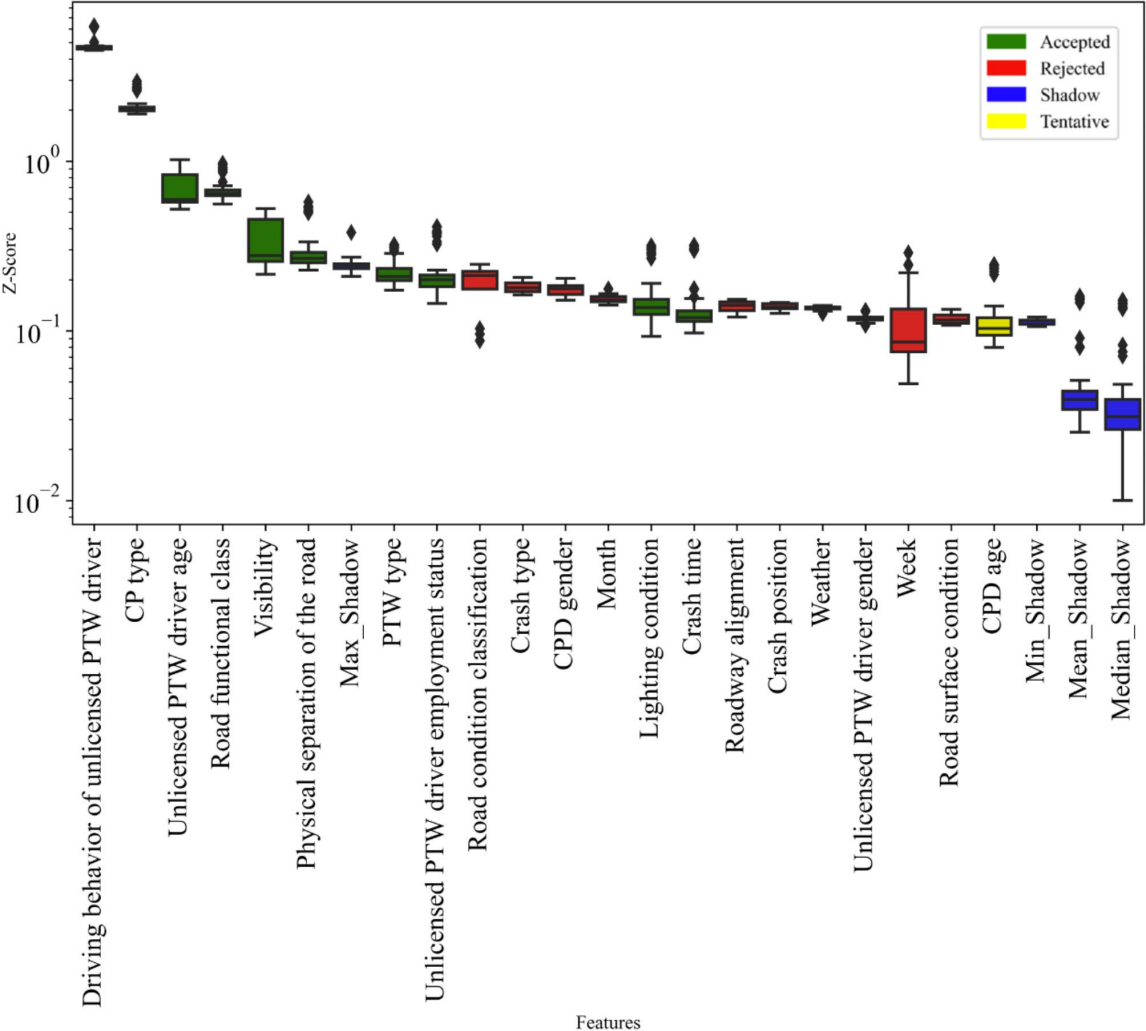
Feature selection.

The unlicensed PTW driver dataset is filtered for features using Boruta-Shap, resulting in an optimal subset of 10 accepted features. The accepted features include: PTW type, Unlicensed PTW driver age, Unlicensed PTW driver employment status, Driving behavior of unlicensed PTW driver, CP type, Road functional class, Physical separation of the road, Crash time, Visibility, and Lighting condition. The green box-plot corresponds to the accepted feature, as shown in the Fig. 4.

**Fig. 5:**
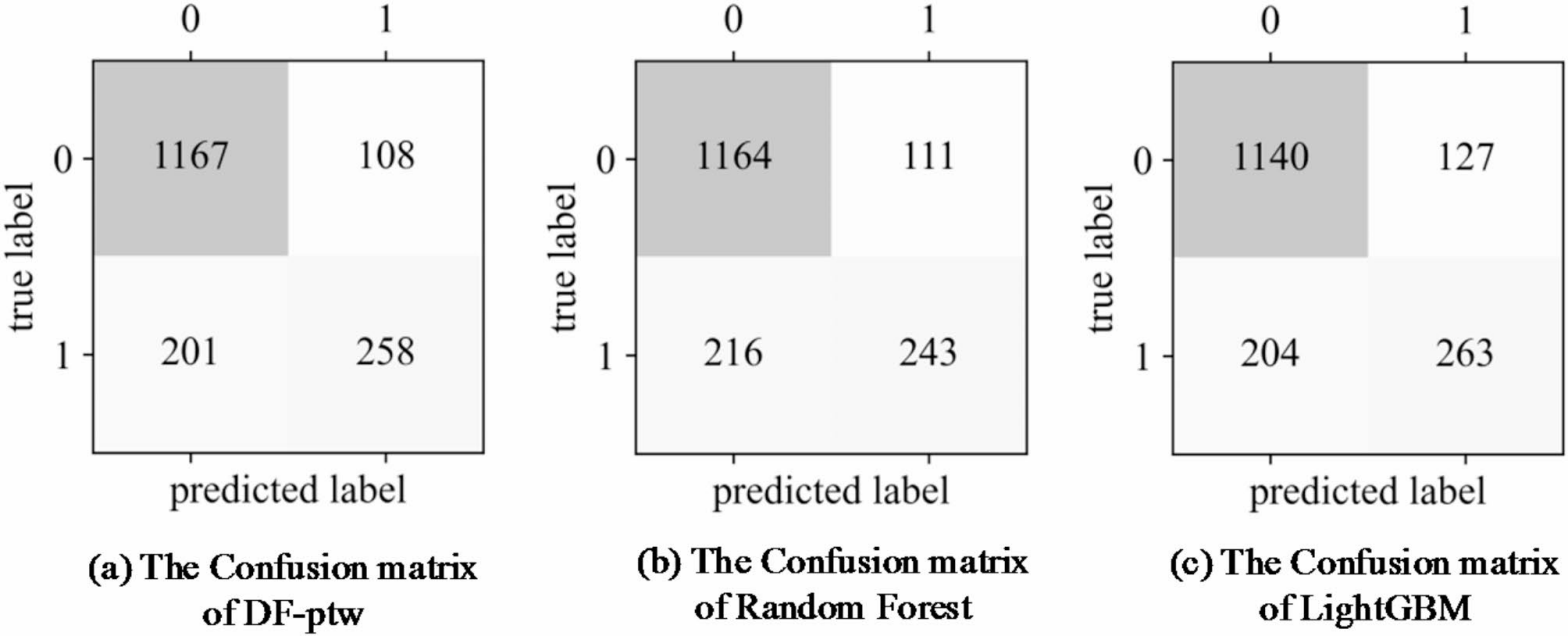
Confusion matrices.

These 10 features are used in the DF-ptw as the optimal subset for predicting injury severity classification. In addition, this study utilizes two predictors-Random Forest and LightGBM for injury severity prediction. The two-dimensional confusion matrices for the three models are shown in Fig. 5.

**Fig. 6:**
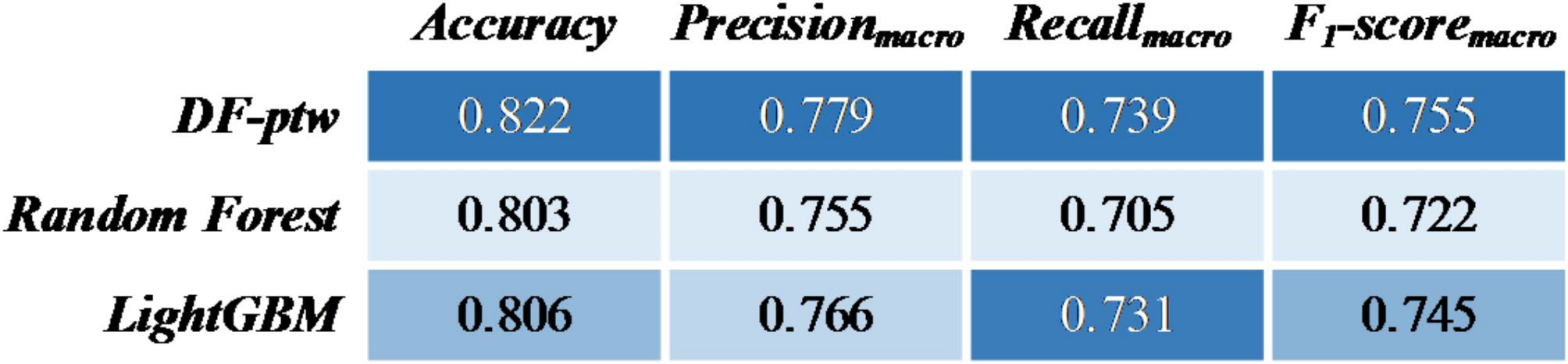
Model comparison.

Model prediction accuracy metrics can be derived from the confusion matrix, as shown in Fig. 6. The DF-ptw outperforms single predictors-Random Forest and LightGBM on the optimal feature subset. The $\:Accuracy$, $\:{Precision}_{macro}$, $\:{Recall}_{macro}$, and $\:{F}_{1}\text{-}{score}_{marco}$ metrics of the DF-ptw model are higher than those of Random Forest and LightGBM.

### Analysis of factors affecting

Since serious and fatal injuries involve the greatest loss of life and property, this study aimed to analyze the significant factors contributing to the SFI. This study categorizes these factors into individual-level and group-level categories. Individual-level factors refer to attributes specific to individual vehicles and drivers, not shared with others (e.g., PTW-related characteristics and CPD-related characteristics). Group-level factors encompass attributes shared by all drivers and vehicles (e.g., Roadway characteristics, Crash characteristics, and Environmental characteristics).

**Fig. 7:**
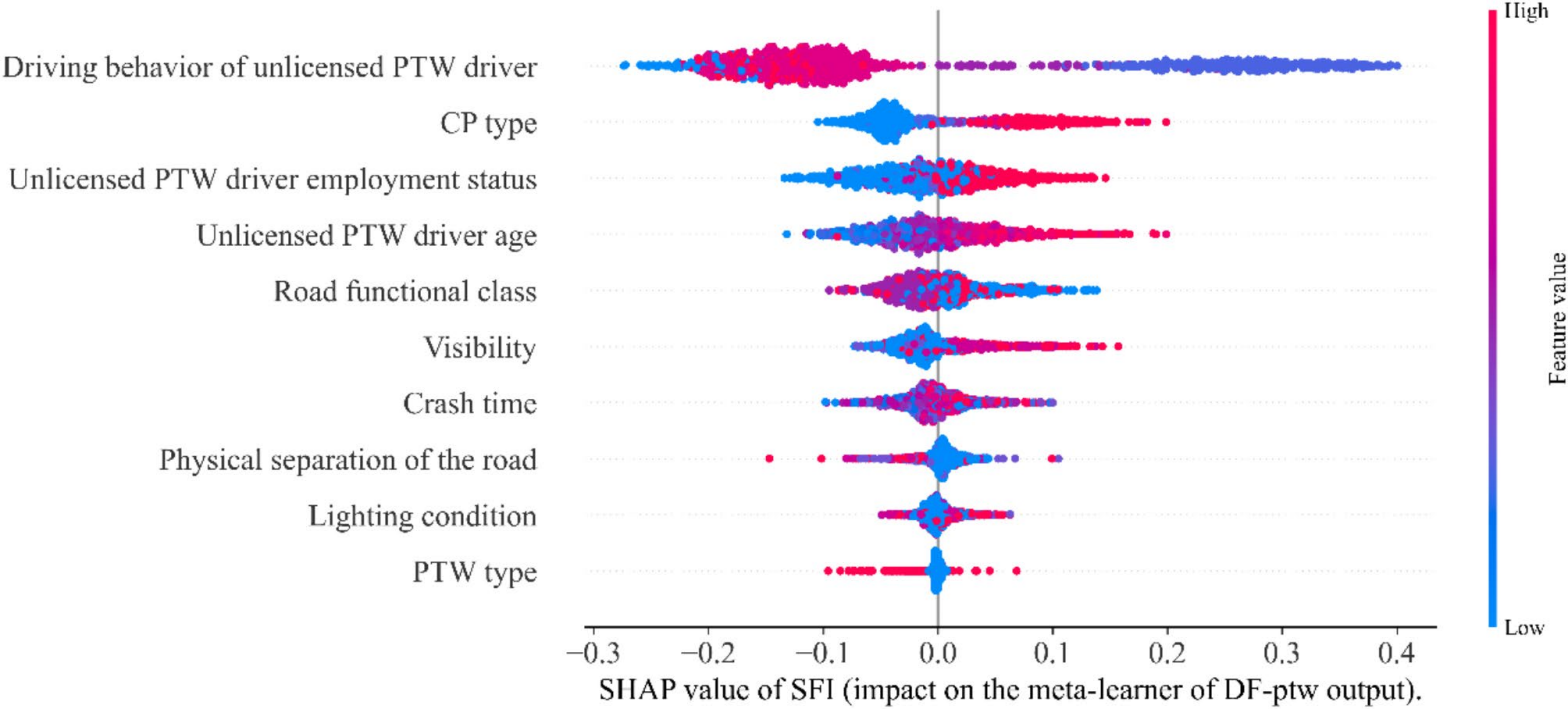
SHAP summary plot of SFI-class.

Different features within the subset vary in their effectiveness in influencing the prediction results. Figure 7 illustrates the feature importance ranking of SFI-class within the optimal subset. The key individual-level factors that significantly influence the probability of the unlicensed PTW driver being SFI in the two-vehicle crash, ranked in order of importance, are: the driving behavior of unlicensed PTW driver, the CP type, the unlicensed PTW driver age, the unlicensed PTW driver employment status, and the PTW type. Additionally, the group-level factors are ranked by importance as follows: the road functional class, the visibility, the crash time, the physical separation of the road, and the light condition.

**Fig. 8:**
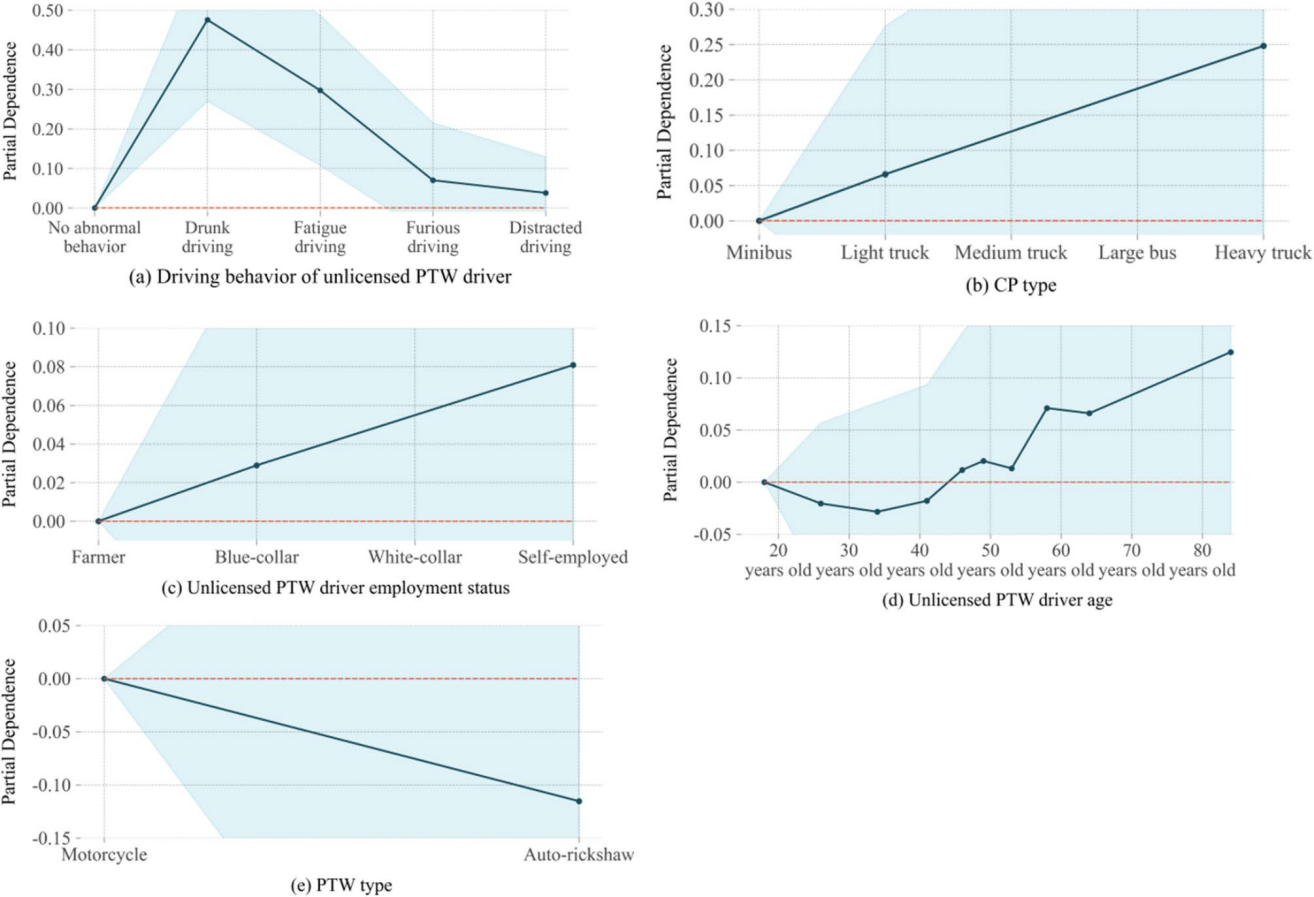
PDP for group-level factors on SFI-class.

**Fig. 9:**
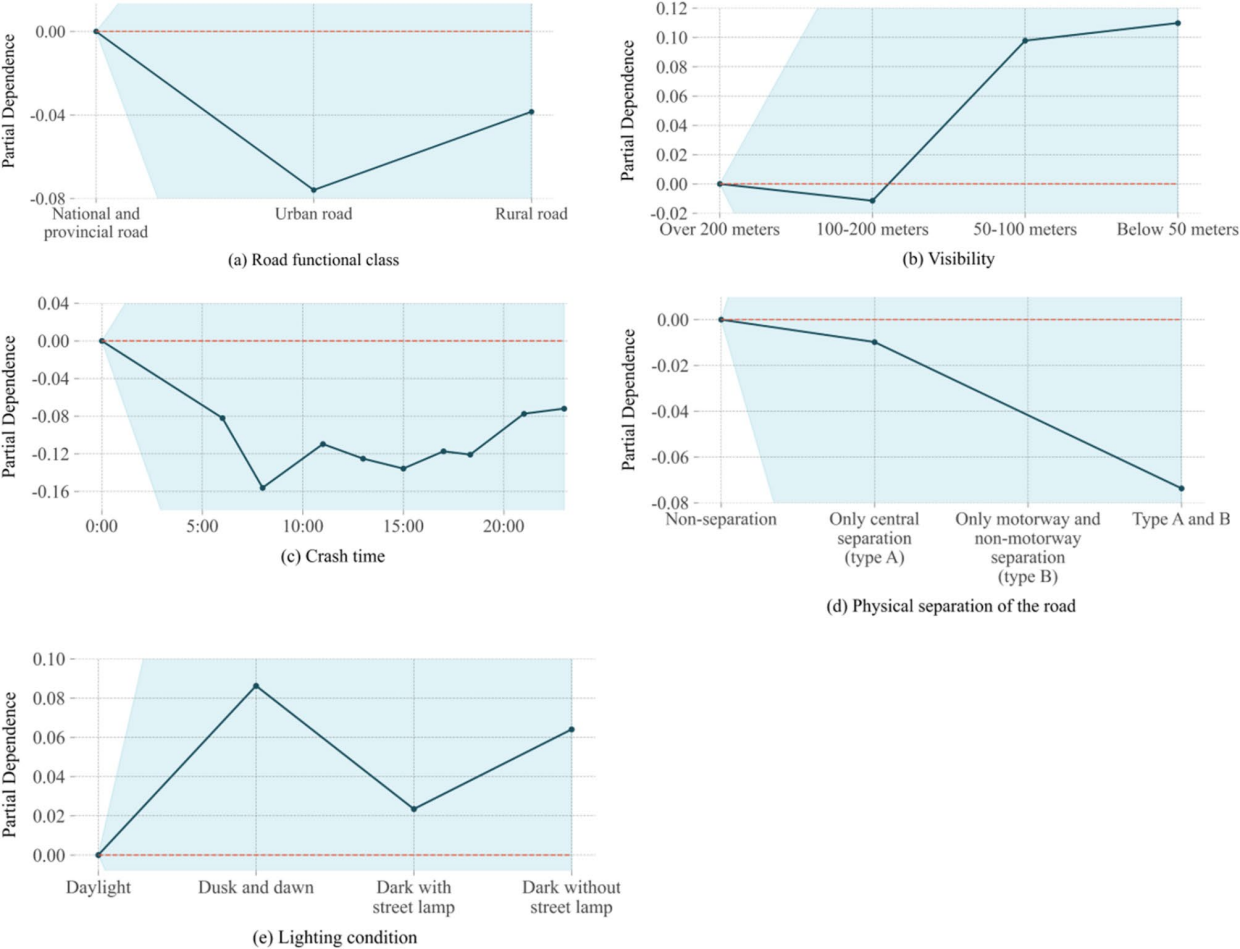
PDP for group-level factors on SFI-class.

In the SHAP summary plot, initial indications of the relationship between the value of a feature and its impact on the prediction are observed. However, to discern the precise nature of this relationship, partial dependence plot must be examined. Figures 8 and 9 show the PDP for individual-level factors on SFI-class and the PDP for group-level factors on SFI-class, respectively. The y-axis represents the change in the predicted value due to the change in the factors, with the blue color indicating the confidence interval.

#### Individual-level factors

(1) Driving behavior of unlicensed PTW driver.

The driving behavior of unlicensed PTW driver pose a significant effect on FSI, as shown in 7. Risky driving behaviors raise the likelihood of involvement in a serious crash^37^. As shown in Fig. 8(a), compared with unlicensed PTW drivers with no abnormal driving behavior, driving under the influence of alcohol increases the probability that drivers suffer SFI in crashes by about 0.5. And, fatigued, furious, and distracted driving each increase the likelihood of unlicensed PTW driver suffers SFI in crashes, the probability of SFI increases by approximately 0.3, 0.08, and 0.05 respectively compared with no abnormal driving behavior. Given the significant impact of alcohol consumption on crash severity, we recommend enhancing the scrutiny of PTW driving qualifications and imposing stricter penalties for unlicensed PTW riders found driving under the influence of alcohol^38^. This approach aims to reduce alcohol-related crash risks and improve road safety.

(2) CP type.

The SHAP value gradually increases as the CP type becomes larger and heavier, in the Fig. 7. Due to the substantial disparities between PTW and large vehicles, unlicensed PTW drivers are more likely to sustain serious injuries when the crash partner is a large vehicle^39^. Compared with crash with the minibus, crashes involving the light truck, medium truck, bus, or heavy truck increase the probability of SFI by approximately 0.03, 0.06, 0.09, and 0.125, respectively, as shown in Fig. 8(b). It is important to note that unlicensed PTW drivers are often seriously injured in crashes involving heavy trucks. Heavy trucks have large blind spots, making it difficult to notice other vehicles when at intersections, turning, or reversing, which can easily lead to serious crashes^40^. Additionally, the weight of these vehicles results in significant inertia, making them difficult to control during sudden emergencies^41^. This leads to longer braking distances and an increased likelihood of crashes. PTW riders are more vulnerable in collisions with larger vehicles, emphasizing the need for enhanced safety measures. To mitigate risks, it is recommended that traffic control authorities promote the installation of blind spot detection systems in heavy vehicles and encourage drivers to slow down or stop at intersections or turns to better observe their surroundings.

(3) Unlicensed PTW driver age.

The SHAP value increases gradually with the unlicensed PTW drivers age, as shown in Fig. 7. Unlicensed PTW drivers aged 20 to 45 tend to have a lower probability of SFI in two-vehicle crashes compared to those aged 18 to 20, possibly due to greater driving experience and emergency response skills. And, older unlicensed PTW drivers have a positive effect on SFI. This has been confirmed by previous studies, which show that older individuals are physically weaker and more likely to be seriously injured in a crash^42^. Changes in predicted values became evident for drivers around older than 53 years, as shown in Fig. 8 (c). And, the probability of SFI in the elderly increases by approximately 0.125 compared with those aged 18 to 20. It is recommended that traffic control authorities intensify checks on the driving qualifications of elderly PTW riders to reduce the number of unlicensed elderly drivers. Additionally, road safety education should be provided to raise safety awareness among elderly drivers.

(4) Unlicensed PTW driver employment status.

The employment status of unlicensed PTW drivers also impacts the injury severities they sustain in two-vehicle crashes.as shown in Fig. 7. Compared to farmer unlicensed PTW drivers, blue-collar, white-collar, or self-employed increase the probability of SFI by approximately 0.03, 0.05, and 0.08, respectively. as shown in Fig. 8(d). Variations in driving styles among unlicensed PTW drivers in different employment status may explain these differences. It is worth noting that some self-employed individuals may perceive obtaining a driver’s license as requiring significant time and financial investment and may not consider it a necessary expense. A study from Sweden found that unlicensed drivers from self-employed families had a higher risk estimate for severe injury than has been reported in other studies^43^. Since self-employed unlicensed drivers are at higher risk due to potential lack of formal training, it is crucial to design education programs that focus on safe driving practices, including the dangers of driving under fatigue or while distracted.

(5) PTW type.

When PTWs crash with four-wheeled vehicles, the type of PTW has a limited impact on crash severity, though some effect is still present. Auto-rickshaw has a SHAP value less than 0, which has a negative effect on SFI, as shown in Fig. 7. Compared to unlicensed auto-rickshaw drivers, unlicensed motorcycle drivers increase the probability of SFI by approximately 0.02, as shown in Fig. 8 (e). The risk of SFI is higher for unlicensed motorcycle drivers due to the lack of safety equipment like helmets and seat belts. The higher risk of severe injury among unlicensed motorcycle riders is primarily due to the lack of safety equipment, such as helmets and seat belts, which increases vulnerability in crashes. It is recommended to enforce policies requiring all PTW riders to use safety gear to reduce injury severity. Additionally, promoting the importance of protective equipment through media campaigns, community activities, and driving schools can raise awareness and emphasize the critical role helmets and other safety gear play in rider safety.

#### Group-level factors

(1) Road functional class.

Traffic volumes, speed limits, and safety facilities vary across different road functional classes, leading to differences in crash severity^44^. According to Fig. 7, the road functional class as a group-level factor poses a significant effect on SFI. National and provincial roads have higher speed limits and traffic volumes compared with urban and rural roads, making serious crashes more likely^45^. Crashes on national and provincial roads increase the probability of SFI by approximately 0.06 compared to urban roads, as shown in Fig. 9(a). There is a huge speed difference between PTWs and four-wheelers on national and provincial roads, which can lead to serious outcomes in crash^46^. Due to poorer infrastructure and road conditions on rural roads, two-vehicle crashes involving the unlicensed PTW driver on a rural roadway increases the probability of SFI by approximately 0.03. Given the increased severity of crashes on national and provincial roads, it is essential to implement stricter speed limits, improved traffic controls, and better road infrastructure maintenance on these roads to reduce crash severity.

(2) Visibility.

Visibility has consistently been a crucial factor in traffic safety. Figure 7 illustrates that low visibility significantly impacts the likelihood of SFI. Compared with visibility greater than 200 m, visibility below 50 m increases the probability of SFI by approximately 0.05, as shown in Fig. 9(b). Studies have shown that low visibility reduces sight distance and greatly increases the risk of traffic crashes, especially on high-traffic roadways prone to rear-end crashes^47^. To address the significant impact of low visibility on crash severity, local authorities should invest in enhanced lighting systems, particularly on roads with limited visibility, such as rural roads or high-traffic areas prone to rear-end crashes.

(3) Crash time.

Factors like traffic volume, visibility, lighting conditions, and driving behavior can change over time. These changes cause varying effects on crash severity across different time periods. The study found that the probability of SFI increased by approximately 0.04 around midnight compared to 7 a.m., as shown in Fig. 9(c). Late-night hours are often associated with a higher incidence of impaired driving due to alcohol consumption or fatigue, both of which are major contributors to severe crashes. And drivers tend to show more risky and aggressive behaviors at night due to reduced traffic volume^48^. Our study shows that the probability of serious injuries increases around midnight, targeted interventions such as increased patrols or temporary traffic restrictions during high-risk hours could be effective in reducing the likelihood of crashes during these times.

(4) Physical separation of the road.

The form of road segregation is crucial for defining user right-of-way and absorbing crash impact energy^49^. As roadway separation improves (from no separation to type A (only central separation) or type B (only motorway and non-motorway separation) to both type A and B), the SHAP value decreases to less than zero, indicating that enhanced separation has a negative effect on SFI as shown in Fig. 7. This means that better separation reduces the likelihood of severe outcomes in crashes^50^. Compared with no separation, both type A and B separation reduces the probability of SFI by approximately 0.04, as shown in Fig. 9 (d). This finding supports the idea that better physical separation of different types of road users significantly mitigates the severity of crashes.

(5) Light condition.

Lighting conditions have a similar effect on crash severity as visibility. The likelihood of SFI increases at dawn, dusk, and during unlit nighttime conditions, as shown in Fig. 9(e). Dazzle can occur at dusk or dawn, while the headlights of oncoming traffic interfere with a driver’s vision at night, especially when there are no streetlights^51^. And, owing to reduced visible range drivers need longer reaction times and space to decelerate when meeting dangerous situations. Enhancing road lighting, particularly at high-risk areas such as intersections or rural roads, would reduce crash risk during night-time driving.

## Conclusion

This study aims to explore the significant influencing factors of Chinese unlicensed PTW drivers in two-vehicle crashes. To achieve this, we constructed a customized Deep Forest Model (DF-ptw) to classify and predict high-dimensional crash data. The Multi-granularity Scanning structure in DF-ptw captures both local and global patterns of the data and enhances the feature representation of the model. Additionally, it enhances the model’s robustness and performance by incorporating multiple types of predictors-Random Forest and LightGBM within the Cascade Forest structure of DF-ptw. The prediction results show that the customized DF-ptw outperforms single-predictor-Random Forest and LightGBM in predicting.

Using SHAP and PDPbox based on the meta-learner of the DF-ptw, key factors influencing two-vehicle crashes involving unlicensed PTW drivers are revealed. Unlicensed drivers who are under the influence of alcohol, unlicensed motorcycle drivers, self-employed unlicensed drivers, and those older than 53 years have a significantly higher risk of sustaining serious injuries in two-vehicle crashes. Additionally, crashes occurring on national and provincial roads, on non-separated roadways, and during late-night hours further increase the likelihood of serious injury or fatality for unlicensed PTW drivers.

In response to these findings, several targeted recommendations are proposed. These include strengthening efforts to scrutinize PTW vehicle driving qualifications to reduce the number of unlicensed drivers. Media campaigns, community events, and driving schools should be leveraged to promote the importance of obtaining a driver’s license and wearing protective gear, with a focus on older drivers and self-employed individuals. Additionally, increasing police presence on the roads to impose stricter penalties on unlicensed PTW drivers is recommended. Traffic authorities should also advocate for the installation of blind spot detection systems on heavy vehicles and encourage drivers to slow down or stop at intersections or turns to prevent crash with PTWs. Improving road lighting in high-risk areas, especially at night, to boost visibility and decrease collision likelihood is also essential. These recommendations aim to reduce the number of unlicensed PTW drivers and mitigate the severity of crashes, ultimately enhancing overall road safety.

There are still some limitations in the current study. The crash data in this study is collected from a province in eastern China. Geographic differences may limit the applicability of these conclusions to northern and western regions. And that the findings may not be directly generalizable to other regions with different traffic conditions, regulations, or demographic characteristics. In future studies, crash severity analysis will focus on mountainous and rural areas in western China, incorporating vehicle data with points of interest (POI) and more detailed data to explore the impact of other relevant factors on crash severity.

## Data Availability

The datasets generated and analysed during the current study are available from the corresponding author on reasonable request.
